# Pre-Drawn Syringes of Comirnaty for an Efficient COVID-19 Mass Vaccination: Demonstration of Stability

**DOI:** 10.3390/pharmaceutics13071029

**Published:** 2021-07-07

**Authors:** Francesca Selmin, Umberto M. Musazzi, Silvia Franzè, Edoardo Scarpa, Loris Rizzello, Patrizia Procacci, Paola Minghetti

**Affiliations:** 1 Department of Pharmaceutical Sciences, Università degli Studi di Milano, Via G. Colombo, 71-Milan, 20133 Milano, Italy; umberto.musazzi@unimi.it (U.M.M.); silvia.franze@unimi.it (S.F.); edoardo.scarpa@unimi.it (E.S.); loris.rizzello@unimi.it (L.R.); paola.minghetti@unimi.it (P.M.); 2 Institute of Molecular Genetics (INGM) Romeo and Enrica Invernizzi, Via F. Sforza, 35-Milan, 20100 Milano, Italy; 3 Department of Biomedical Sciences for Health, Università degli Studi di Milano, Via G. Colombo, 71-Milan, 20133 Milano, Italy; patrizia.procacci@unimi.it

**Keywords:** aggregates, compatibility, COVID-19, DLS, gel electrophoresis, in-use stability, mass vaccination, NTA, TEM

## Abstract

Moving towards a real mass vaccination in the context of COVID-19, healthcare professionals are required to face some criticisms due to limited data on the stability of a mRNA-based vaccine (Pfizer-BioNTech COVID-19 Vaccine in the US or Comirnaty in EU) as a dose in a 1 mL-syringe. The stability of the lipid nanoparticles and the encapsulated mRNA was evaluated in a “real-life” scenario. Specifically, we investigated the effects of different storing materials (e.g., syringes vs. glass vials), as well as of temperature and mechanical stress on nucleic acid integrity, number, and particle size distribution of lipid nanoparticles. After 5 h in the syringe, lipid nanoparticles maintained the regular round shape, and the hydrodynamic diameter ranged between 80 and 100 nm with a relatively narrow polydispersity (<0.2). Samples were stable independently of syringe materials and storage conditions. Only strong mechanical stress (e.g., shaking) caused massive aggregation of lipid nanoparticles and mRNA degradation. These proof-of-concept experiments support the hypothesis that vaccine doses can be safely prepared in a dedicated area using an aseptic technique and transferred without affecting their stability.

## 1. Introduction

The first vaccine approved in the EU and the US for the prevention of COVID-19, caused by the SARS-CoV-2 virus, is based on mRNA strands packaged in neutrally charged lipid-based nanoparticles (LN). This formulation is commercialized as a deep-frozen concentrate for dispersion (Pfizer-BioNTech COVID-19 Vaccine in the US or Comirnaty in EU), which is diluted (upon thawing) just before injecting a 0.3-mL dose intramuscularly.

The success of this innovative treatment is based on the use of (i) highly modified and purified in vitro transcribed mRNA enabling a high protein expression; (ii) the delivery vehicle, i.e., LN, which improves mRNA stability and promotes endosomal escape upon cellular uptake [1].

Being the very first mRNA-based therapeutic to receive approval by the FDA and EMA and the relative speed at which the vaccine has made it on the market, limited information about its stability can be retrieved in the “Summary of Product Characteristics” (SPC) or literature [2,3,4]. As a consequence, in early 2021, the vaccination campaign had hard tested the operativity of logistic and healthcare systems, as the vaccine concentrate has to be prepared and administered in very short periods since it has to be maintained at 2–8 °C for a maximum of 5 days upon thawing, and diluted and administered within 6 h [5,6]. This has been particularly critical considering that no doses have to be wasted due to mishandling procedures or a delay in administration to boost mass vaccination.

Very recently, the EMA authorized an expansion of undiluted vaccine shelf-life at 2–8 °C up to 1 month [4]; other “real life” scenarios should be considered to define the stability of the mRNA vaccine. Indeed, doses are distributed from hubs, where vials are stored at −90/−60 °C, to the points of vaccination (e.g., vaccination hubs, community pharmacies). In this context, it cannot be excluded that the vaccine doses in a vial are administered in the same setting, but they could be transported by healthcare professionals to reach a domiciliary setting. The unperforated product can be transported at 2–8 °C either in two trips, each one having either a duration of up to 6 h or for a maximum of 12 h in one sitting [7]. Again, road transportation needs special care to ensure that the vaccine quality is not impaired by environmental conditions, such as temperature and shocking [8]. To the best of our knowledge, safe transport of the diluted vaccine is not currently supported by relevant data [9].

Finally, the compatibility “with commonly used commercially available administration components” is quoted in the EMA or FDA assessment reports [5,6]. Since January 2021, the USP has published and updated a COVID-19 Vaccine handling toolkit to assist those who handle these vaccines in challenges that may arise during their preparation. This document is based on the scientific and professional expertise of the members of the expert committees of the US Pharmacopeia (USP) [10]. Nevertheless, no data on the environmental factors controlling and limiting vaccine stability in the syringe are publicly available, as of the writing of this manuscript. This lack of information seems particularly critical taking also into consideration that researchers of the European Commission’s Joint Research Centre (JRC) demonstrated that nanosystems’ retention in the syringe might be a critical drawback affecting the reproducibility of dosing of several types of nanosystems (e.g., inorganic nanoparticles, exosomes, protein-based nanoparticles, nanocrystal) in in vivo studies [11].

In an attempt to fill the gaps of background information, we investigated the stability of LN and mRNA of Comirnaty batches stored in either glass vials or 1-mL plastic syringes (made of poly(propylene) or poly(carbonate)), both kept in cold chain (i.e., 2–8 °C), or room temperature (25 °C) over 5 h. The impact of road transportation for 30 km in a suburban area using a temperature stabilizing medium was explored. The physical stability of Comirnaty was assessed by dynamic light scattering (DLS) and nanoparticle tracking analysis (NTA), while mRNA stability was determined by gel electrophoresis. The first method was selected since it is included in the quality control test performed by the manufacturer, as reported in the European Public Assessment Report (EPAR) published on the EMA portal [5]. Even though DLS is a fast and robust quality control method to determine the size and to detect nanoparticle aggregation, it has a low particle size resolution, and smaller particles may be under-represented in the case of polydisperse samples. This is the reason why we also proposed the use of NTA, which provides quantitative data (i.e., mean diameter, D_90_, nanoparticles concentration) that can be also analyzed from a statistical point of view.

## 2. Materials and Methods

### 2.1. Materials

In agreement with the instruction on the authorized package leaflet, the dilution of the concentrated vaccine (Comirnaty) was carried out in aseptic conditions in hospital pharmacies of institutions located in Lombardy (Italy). Afterwards, leftovers of three different commercial vaccine batches (i.e., Batch EJ6796, #1; Batch EL1484, #2; EP2166, #3) were collected for experimental purposes. Leftovers filled in single-use syringes made of poly(propylene) and poly(carbonate) or in original glass vials were transported to the laboratory for analysis by road in a suburban area for 30 km using a temperature stabilizing medium. All samples were stored at 2–8 °C until use unless specified.

### 2.2. Stress Tests

Aggregates can be generated by a variety of stress conditions which can occur during various stages of the manufacturing process, shipping, and administration, and/or accelerated by various external factors such as heating, mechanical stress, formulation changes, and storage. The control of the small particulate is necessary for ensuring the safety and efficacy of drug products. For this reason, different accelerated stress conditions were generated to assess the stability of the diluted solutions of LN. For mechanical stress, LN samples were manually pumped 50 times through a disposable 2 mL single-use syringe. Moreover, leftovers in vials were vortexed for 2 min at 1200 rpm. For thermal stress, leftovers in vials and syringes were incubated at 25 ± 1 °C and 40 ± 1 °C for 24 and 5 h, respectively.

### 2.3. Dynamic Light Scattering

The Z-average diameter (D_h_) and the polydispersity index (PDI) of samples were evaluated by photon correlation spectroscopy using a dynamic light scatter (DLS, Zetasizer Nano ZS, Malvern Instrument, Malvern, UK), equipped with a backscattered light detector, operating at 173° and 25 °C. The results calculated using the Dispersion Technology Software (Malvern Instruments, Malvern, UK) are reported as intensity distribution.

### 2.4. Nanoparticle Tracking Analysis

Particle size distribution and concentration were analyzed by a nanoparticle tracking analysis (NTA) using a NanoSight NS300 (Malvern Panalytical, Malvern, UK) equipped with a blue laser (404 nm, 70 mV) and sCMOS camera. Before the analysis, the samples were diluted in physiologic sterile solution to reach the ideal particle concentration range in terms of particles/frame (20–120 particles/frame). Temperature was held constant at 25 °C during the experiment, and for each sample, five 60 s videos were recorded and, subsequently, analyzed using NTA software 3.0 (NanoSight, Malvern Panalytical, Malvern, UK).

### 2.5. Transmission Electron Microscopy (TEM)

Aliquots of 5 μL of the leftover liquid from diluted vaccines within the original glass vials were absorbed on Formvar-coated single-slot grids. After 2 min, the excess solution was removed with filter paper. Grids were air-dried, stained with freshly filtered 2% aqueous uranyl acetate for 7 min, washed in MilliQ water, and allowed to dry. Grids were examined under a Zeiss EM 10 electron microscope (Gottingen, Germany) at 80 kV voltage.

### 2.6. mRNA Integrity and Quantification

mRNA integrity has been assessed by agarose gel electrophoresis. Briefly, a 1.8% (*w*/*v*) agarose (Merck, Roma, Italy) solution in (1×) Tris-Borate-EDTA (TBE) buffer (pH 8, ThermoFisher, Rodano, Italy) with ethidium bromide has been firstly boiled and then cooled down to 60 °C, before pouring it within the gel cassette. The vaccine samples (equivalent mRNA amount: 50, 20, and 10 ng) were mixed with the gel loading buffer (95% formamide, 18 mM EDTA, and 0.025% SDS, Xylene Cyanol, and Bromophenol Blue, ThermoFisher, Rodano, Italy) and then diluted with DEPC-water (ThermoFisher, Rodano, Italy) to a final volume of 40 µL. Samples were loaded in the wells and the gel run at 8 V/cm in (1×) TBE running buffer, and finally visualized with a UV-transilluminator. The mRNA quantification was performed by UV-Vis (Nanodrop, ThermoFisher, Rodano, Italy).

### 2.7. Data Analysis

Tests for significant differences among means were performed by the one-way ANOVA followed by Bonferroni–Holm post-analysis (Daniel’s XL Toolbox for Microsoft excel, D). Differences were considered significant at the *p*-value < 0.05.

## 3. Results and Discussion

Before the need to control the COVID-19 pandemic, LN represented a leading technology for the systemic delivery of siRNA (i.e., Onpattro) [12]. This drug delivery system consists in multicomponent nanoparticles composed of ionizable amino lipids, phospholipids, cholesterol, and a polyethylene glycol-lipid conjugate (PEG-lipid). The ionizable amino lipid plays a principal role in siRNA transfection, mediating cytosolic delivery of the siRNA through facilitated endosomal escape after LN endocytosis. Neutral lipids, such as phospholipids and cholesterol, are selected to modulate the fluidity and phase behavior of the LN, whereas PEG-lipids are utilized to improve particle plasmatic half-life and systemic exposure [13]. In the case of mRNA-based vaccines, LN have a relevant role in (i) protecting mRNA from the possible enzymatic degradation and (ii) masking the negative charge which would prevent passive internalization. The common ground of siRNA and mRNA delivery systems is the presence of ionizable amino lipids, termed asymmetric ionizable amino lipids. The primary distinguishing features of these novel excipients are the unequal lengths of their unsaturated hydrocarbon chains and their corresponding low molecular weight. These features facilitate the metabolism of the lipids in vivo, resulting in faster lipid plasmatic half-life and imparting significant improvements in the product tolerability. Asymmetric amino lipids further favor the adoption of disruptive non-bilayer structures, facilitating cargo release. At the same time, asymmetric LN present a significant challenge in pharmaceutical developability, namely physical instability limiting extended shelf life [14]. It has been recently demonstrated that, contrary to typically ascribed modes of nanoparticle instability (i.e., aggregation and fusion), the mechanism of LN instability is Ostwald ripening which is predominantly mediated by the low molecular weight and molecular architecture of asymmetric amino lipid components [14]. At the same time, due to the novelty of this drug delivery system, a paucity of information in the post-marketing phase is available about the possible causes of instability and their effect on other quality features of LN. 

It is noteworthy that factors affecting LN and mRNA stability during shelf-life have been recently in-depth reviewed by Schoenmaker and the co-authors [2]. It was underlined the importance of adopting multiple analytical techniques for product characterization to meet the intrinsic complexity of such nanosystems. However, despite scientific findings on different nanosystems suggesting that their retention in syringes might be a quality issue [11], the in-use stability of LN and/or their compatibility with administration devices has not been documented yet. To fill this gap, LN were primarily characterized by TEM, DLS, and NTA, as these complementary techniques can provide evidence on variations in morphology, size, and size distribution which can drastically change the formulation features, including the dose-to-dose reproducibility, the fate, and the biodistribution of the particles upon injection [3,15,16]. 

**Figure 1:**
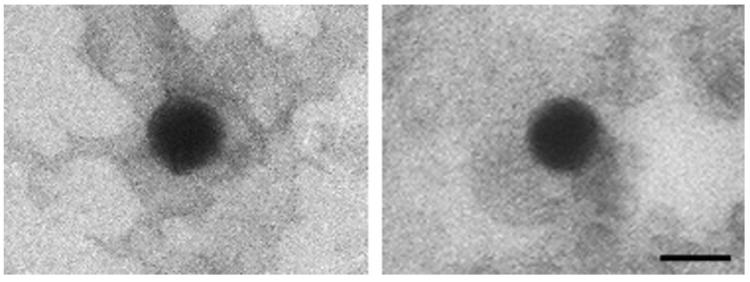
Representative TEM photomicrographs showing nanoparticles from the diluted concentrated vaccine. Scale bar: 100 nm.

TEM analysis of the leftover in the original glass vial showed nanoparticles with an electron-dense core, a uniform size (≈100 nm), and a regular round shape (Figure 1).

**Figure 2:**
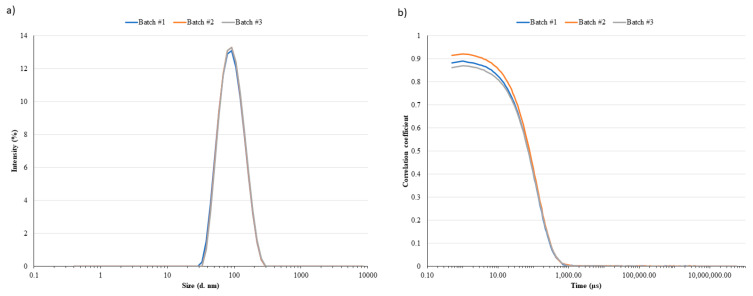
Aliquots of Comirnaty from three different batches were analyzed by Dynamic Light Scattering (DLS) to determine the hydrodynamic diameter based on the intensity distribution curve. (**a**) Samples were monodispersed, and the height of the peak signal overlapped. (**b**) These considerations were also supported by the correlogram plots.

As reported in Table 1, leftovers’ LN analyzed by DLS resulted in D_h_ ranging between 80 and 100 nm with a relatively narrow polydispersity (PDI < 0.2, Figure 2a). Small differences, but not statistically significant, were observed between the two batches (#1 vs. #2; *p*-value = 0.2476). The correlogram functions (Figure 2b) supported the consistency of collected data. Indeed, although the multiple scattering fluctuations (correlation intercept < 1) were due to a slightly high LN concentration in the sample, the three batches showed a superimposable decay of the correlation function confirming the inter-batch homogeneous LN size distribution. The particle size distribution derived from NTA analyses and expressed in terms of D_10_, D_50_, and D_90_ was superimposable for batch #1 and #2 (D_10_: 56 ± 1 nm and 59 ± 4 nm; D_50_: 78 ± 5 nm and 78 ± 4 nm; D_90_: 124 ± 9 nm and 124 ± 6 nm for batch #1 and #2 leftovers, respectively). These data were expected to some extent considering that most of the nanomedicine products approved and available on the market are stored in vials as aqueous dispersions and are stable at 2–8 °C for all shelf-life periods. As an example, Onpattro is a concentrate for a solution for an infusion stored in refrigerated conditions (2–8 °C) [12]. Among all approved liposomal medicinal products, only two are available as freeze-dried powders to improve the product stability.

On the contrary, studies on the physical stability of nanoparticles in disposable syringes are not available in the literature. At the time of writing, only few data were collected on radiolabeled nanosystems and exosomes demonstrating a certain degree of particle retention into plastic insulin-type syringes [11]. Comirnaty LN from different batches instead were physically stable in disposable syringes, regardless of the device material, storage temperature, and road transportation since no sign of aggregation or adsorption was detected at 2–25 °C over 24 h (Table 1 and Table 2).

Indeed, the D_h_ and PDI of LN contained in syringes of different materials remained within ±5% of the initial vials in all in-use conditions and in samples stored for 24 h at different temperature concentrations (Table 2). Moreover, although a slight D_h_ increase of LN contained in syringes was observed in comparison to those in glass vials, all values remained within the observed inter-batch variability. Such negligible variations in the size distribution in LN syringes suggested no phenomena related to the colloidal instability throughout the storage period. Indeed, throughout the storage period, the percent coefficient of variations of both parameters (D_h_, PDI) remained within the ranges observed for the LN suspensions contained in the vials (±5%; Table 1). Analogously, no significant variations in particle concentration were observed, ruling out the formation of new populations of particles during the storage in the syringe. This evidence seems to suggest that, unlike radiolabeled nanosystems and exosomes [11], LN are not retained significantly in the syringes up to one day regardless of the storage temperature (Table 1 and Table 2).

**Figure 3:**
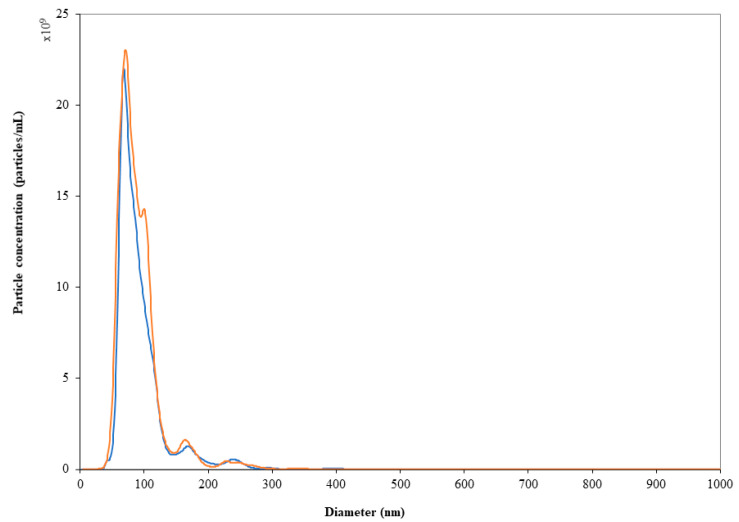
Particle size distribution registered by NTA for vial leftover which underwent (blue line) or not (orange line) road transportation. Any significant variations in particle distribution can be ascribed to the road transportation of vial leftover.

Road transportation did not affect either particle size or polydispersity of the LN dispersions (Table 2). NTA results agreed with DLS data: the particle size distribution and nanoparticle concentration did not change significantly (*p* > 0.05) after storage in syringes at both temperatures over 24 h as well as after road transportation (Figure 3). It is worthy of note that prolonging the storage period up to 10 days from dilution did not affect the physical stability of LN. In fact, although a slight but significant increase in the particle counts was found (Table 2), the mean particle diameter and D_90_ remained unvaried (*p* > 0.05), ruling out the hypothesis of particle aggregation during storage.

However, the effect of important shocking and vibrations needs to be carefully considered since vortexing and syringe mechanical stress caused a significant increase in size and polydispersity (Table 2).

The integrity and stability of mRNA in LN were assessed over time (up to 5 h) through agarose gel electrophoresis of samples stored either in the glass vials (the original sample holder) or within the plastic syringes. The first set of experiments aimed to quantify mRNA loading by UV-Vis based nanodrop which confirmed a very consistent and reliable mRNA concentration in the range of 178 to 185 ng/µL (Table 3) among different samples.

The 260/280 and 260/230 ratio analyses might indicate the presence of extra components such as proteins and/or salts. Despite by the heterogeneous nature of the samples, whose components have not been purified before the analyses, both ratios remained constant, confirming the lack of interaction with the selected packaging materials.

**Figure 4:**
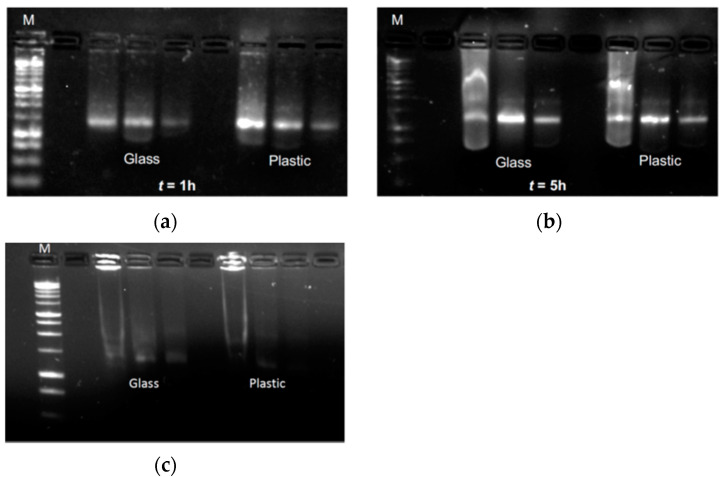
Gel electrophoresis analyses over mRNA samples after 1 (**a**) and 5 h (**b**). As a positive control, a sample of LN mechanical stressed for loading on agarose gel (**c**) For each experimental group (i.e., samples stored in glass vials or plastic syringes), vaccine was further diluted to get 3 different amounts of mRNA, namely 50, 20, and 10 ng (left-to-right lanes).

Afterwards, aliquots of the diluted vaccine were incubated in either the original container (i.e., the glass vial) or the plastic syringe at 2–8 °C for 1 or 5 h, before being characterized by agarose gel electrophoresis to check for potential degradation. Regardless of the storage condition, the gel documentation shows the presence of the same mRNA band for each concentration tested (Figure 4), with the expected drop in the detected signal due to the decrease in mRNA mass loaded. However, when LN were exposed to mechanical stress (i.e., vortexing at 1200 rpm for 2 min), the mRNA underwent complete degradation. This is demonstrated by the lack of the mRNA band, and by the presence of an evident smear through the run lanes (Figure 4). Despite the unavailability of raw materials that could function as a control, the comparison among this set of data suggests that the mechanical stress impairs the physical stability of the LN that also results in the degradation of the mRNA payload. This evidence supports the hypothesis of the strong stability and the integrity of mRNA in the proposed “in-use” condition.

## 4. Conclusions

The pandemic has accelerated the development of innovative vaccine technologies that could be used also for other unmet clinical needs. As the progress has also involved the regulatory pathway and GMP manufacturing, the market access of “second generation” mRNA-based drug delivery systems would be granted at a higher pace. As a key performance criterion in the vaccine formulation, colloidal stability and encapsulation are quality attributes able to significantly dictate their efficacy. Indeed, the unique combination of size and composition rules their biodistribution pattern, immunostimulatory profile, and tolerability. However, the stability of mRNA therapeutics and relative specifications to define their quality attributes are still a challenging area in pharmaceutical development. Moreover, the scientific community agrees that proper storage, handling, and transportation are critical since failures can potentially reduce potency, leading to an inadequate immune response in patients. Restrictions (i.e., storage of frozen samples at −70 °C, or use of thawed samples within 5 days) to preserve LN integrity can be applied during early clinical studies, but as a step forward, an easier distribution should be also taken to develop a highly promising platform, and to assess their stability in devices used for the administration (e.g., syringes).

This proof-of-concept study demonstrated the colloidal stability of mRNA-based LN in syringes made of different materials when subjected to mechanical and thermal stresses of relatively low intensity and short duration. In the context of the current pandemic situation, these data support the hypothesis that an aseptic technique (preferentially in a dedicated area or room) should be utilized to preserve the microbiological stability of single doses before being transported to the point of administration. In a more comprehensive perspective, the experimental set-ups and outcomes of this work have advanced the understanding of the features of these innovative delivery systems which would accelerate the pace towards new clinical applications.

## 5. Limitation of the Study

It is worth mentioning that both the gel electrophoresis and nanodrop-based quantification experiments were carried out with only commercially available material. No control samples are available for analyses, such as the empty (unloaded) LN or the free mRNA (not encapsulated). In this framework, we cannot exclude, for example, a contribution in the absorption measurements (nanodrop) by the LN. Similarly, the gel electrophoresis might under-represent any potential degraded mRNA. Nevertheless, and to the best of our knowledge, it is impossible to gather such control material, at least by the time of this article writing.

## Figures and Tables

**Table 1:** Impact of storage and syringe material on the particle size distribution of LN expressed as the ratio of hydrodynamic diameter (D_h_), which was calculated on the main peak of DLS data expressed in intensity, and polydispersity index (PDI) at different sampling times.

Storage Conditions	Vial/Syringe	Time (h)	D_h_ (nm)	PDI	LN/mL (10 ^11^ Particles/mL)
2–8 °C	Leftover in vial #1	0	93 ± 5	0.154 ± 0.005	11.5 ± 0.7
	Leftover in vial #2	0	98 ± 4	0.166 ± 0.010	9.3 ± 1.2
	Leftover in vial #3	0	91 ± 4	0.155 ± 0.029	6.6 ± 0.9
	Poly(propylene)	0	100 ± 3	0.142 ± 0.008	11.5 ± 0.7
		5	96 ± 3	0.143 ± 0.030	10.9 ± 1.8
		24	99 ± 3	0.154 ± 0.005	11.8 ± 1.8
	Poly(carbonate)	0	100 ± 4	0.160 ± 0.017	9.3 ± 0.4
		5	102 ± 6	0.152 ± 0.019	13.0 ± 1.3
		24	101 ± 3	0.141 ± 0.005	8.3 ± 0.6
25 °C	Poly(propylene)	0	100 ± 3	0.142 ± 0.008	7.0 ± 0.6
		5	96 ± 3	0.142 ± 0.010	8.1 ± 1.0
	Poly(carbonate)	0	91 ± 4	0.155 ± 0.029	7.0 ± 0.9
		5	n.d.	n.d.	10.7 ± 4.0

**Table 2:** Impact of storage and mechanical stress on the particle size distribution of LN expressed as the ratio of hydrodynamic diameter (D_h_), which is calculated based on the main peak of DLS data, and polydispersity index (PDI) before and after the applied experimental condition. DLS: Legend of symbols expressing a variation <10% (=); 10–25% (+); >25% (++); NTA: *p*-values of Student’s t-test of particles before and after the applied experimental condition. n.d.: not determined.

Conditions of Thawed Vaccine		D_h,f_/D_h,i_	PDI_f_/PDI_i_	LN/mL
In-use	Stored in syringe at 2–8 °C for 5 h	0.96 (=)	1.00 (=)	*p* = 0.86
	Stored in syringe at 25 °C for 5 h	0.96 (=)	1.00 (=)	*p* = 0.17
	Road transportation of syringe at 2–8 °C	1.02 (=)	1.09 (=)	*p* = 0.10
Stressed	Stored in vial at 2–8 °C for 10 days after thawing	1.01 (=)	1.04 (=)	*p* < 0.01
	Stored in syringe at 2–8 °C for 24 h	0.99 (=)	1.04 (=)	*p* = 0.36
	Stored in syringe at 25 °C for 24 h	1.01 (=)	1.11 (=)	*p* = 0.09
	Stored in syringe at 40 °C for 5 h	0.96 (=)	1.20 (+)	n.d.
	Mixing different vial overfill	1.03 (=)	1.19 (+)	n.d.
	Mixing by vortex for 2 min	1.19 (+)	2.64 (++)	n.d.
	Syringe mechanical stress (40 cycles)	11.37 (++)	7.03 (++)	n.d.

**Table 3:** mRNA quantification and sample purity analyses by UV-vis spectrophotometry (n = 5 measurements).

Sample	ng/µL	260/280	260/230
Glass vial	185 ± 2	1.65 ± 0.11	0.82 ± 0.08
Plastic syringe	178 ± 5	1.63 ± 0.13	0.84 ± 0.05

## Data Availability

Not applicable.
